# Enhancing Solid Booster Utilization in Redox-targeted Flow Batteries with Non-fluorinated Binders

**DOI:** 10.1021/acsmaterialslett.5c01591

**Published:** 2026-02-03

**Authors:** Julia Lorenzetti, Paweł P. Ziemiański, Cédric Kupferschmid, David Reber

**Affiliations:** † Empa, Swiss Federal Laboratories for Materials Science and Technology, 8600 Dübendorf, Switzerland; ‡ École Polytechnique Fédérale de Lausanne, Institute of Materials, 1015 Lausanne, Switzerland

## Abstract

Redox-targeted flow batteries (RTFBs) are promising for large-scale energy storage but suffer from poor solid booster utilization. This study examines how binder selection affects the reaction rate between a LiFePO_4_/FePO_4_ solid booster composite and a dissolved [Fe­(CN)_6_]^4–/3–^ redox mediator. The porosity and hydrophilicity of LiFePO_4_ composites correlate with booster utilization, determined by galvanostatic cell cycling and by *in situ* UV–Vis spectroscopy. Compared with state-of-the-art polyvinylidene difluoride composites, booster pellets containing non-fluorinated, biodegradable polycaprolactone or cellulose acetate binders exhibit up to 175% higher LiFePO_4_ conversion rates and improved capacity utilization at cycling rates up to 10 mA cm^–2^. Solid-material utilization directly correlates with binder hydrophilicity, establishing it as a key design parameter for RTFBs and offering a straightforward path toward more efficient and non-fluorinated booster formulations.

## Graphical abstract

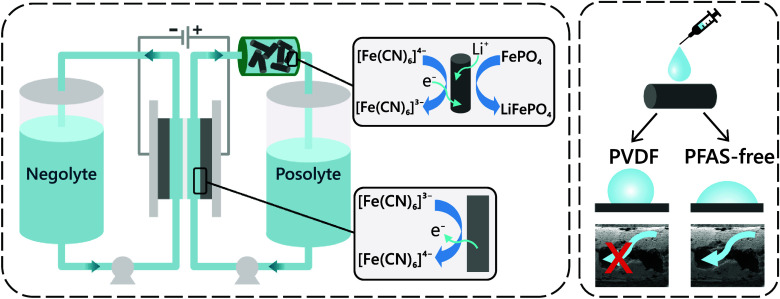

The global expansion of renewable energy demands large-scale energy storage to bridge the spatial and temporal mismatch between energy supply and demand.^1,2^ Aqueous redox flow batteries (RFBs) have emerged as a promising solution for grid-scale storage, in part because of their scalability and inherent non-flammability. In RFBs, the redox-active species are dissolved in liquid electrolytes in reservoirs outside the cell stack, which allows the power and the storage capacity of the battery to be scaled independently. While the power is dictated by the total electrode area in the cell stack, the storage capacity scales with the concentration and the volume of the electrolyte in the external reservoirs. This unique architecture allows for flexible system design and long lifetimes because the electrodes do not undergo any phase transitions upon cycling.^1,3,4^ One of the major challenges in RFB research is low volumetric energy density which is mainly limited by the solubility of active species in the aqueous electrolytes. The volumetric energy density of commercial vanadium-based RFB systems reaches 20–35 Wh L^–1^, an order of magnitude lower compared to LIBs.^3,5^ Increasing the energy density of RFBs could open new use cases, such as indoor use with limited space availability, and could simultaneously reduce costs and decrease electrolyte tank size.^6^

To address this issue, the concept of redox targeting has been applied to RFBs to drastically increase energy density^7–10^ while avoiding high viscosity and slow kinetics that are common for highly concentrated electrolytes.^11,12^ A redox-targeted flow battery (RTFB) confines a solid active material, the solid booster, within the electrolyte reservoir where it regenerates the active material in the solution, the redox mediator, through a chemical redox reaction.^13^ Because of its condensed state, the charge carrier concentration in the solid booster is an order of magnitude higher than what is practically achievable with active species in solution, e.g., 22.8 M for lithium iron phosphate (LFP).^14^ While power and capacity remain decoupled, the capacity of the RTFB thus relies on the energy density of the solid active material rather than electrolyte volume or concentration. Although the booster could in principle be dispersed directly in the electrolyte, high pumping losses and complex fluid dynamics render slurry electrolytes very challenging.^15,16^ In RTFBs, the solid booster is often confined in the form of a composite that typically consists of the active material, a binder such as polyvinylidene difluoride (PVDF), and sometimes a conductive additive like carbon black.^17^ Since the introduction of the concept by Wang et al.^18^ and its more recent demonstration in an aqueous RFB by Zanzola et al.,^19^ a range of materials have been assessed as solid boosters, among them Prussian blue and its analogues,^20–22^ LFP,^23–25^ metal hydrides and hydroxides,^26,27^ and organic polymers.^19,28,29^

Throughout the literature, RTFB systems exhibit low booster utilization and limited rate performance.^30^ For example, in a system with LFP/FePO_4_ as solid booster and [Fe­(CN)_6_]^4–/3–^ as mediator, the full capacity of the LFP added was only accessible at 0.25 mA cm^–2^ while at 1 mA cm^–2^ the utilization dropped to 45%.^23^ Such current rates are orders of magnitude lower than the hundreds of mA cm^–2^ typically applied in conventional RFBs. Recent reports have identified reactor design,^22,31^ morphology and packing of the booster material,^22–24^ and matching redox potentials of booster and redox mediator^23,28,32,33^ as critical factors to increase the efficiency of the redox targeting reaction. The porosity of the booster composite has also been recognized as a parameter influencing the reaction rate with the mediator,^23,24,34^ but prior work has not systematically compared porous and non-porous boosters, and the interplay between composite formulation and the booster-mediator interface remains underexplored.

Interestingly, most of the reported RTFB systems use PVDF, at concentrations from 5 to 50 wt%,^22,24,28,32^ as a binder in the booster composite, although its strong hydrophobicity presumably hinders the penetration of the aqueous electrolyte into the booster pellet. As such, the wettability of inorganic solid boosters has not been addressed, even though it likely affects electrolyte penetration and booster utilization. The increasingly strict regulation on per- and polyfluoroalkyl substances (PFAS) further motivates the replacement of PVDF with a non-fluorinated alternative offering comparable performance.^35^

Here, we investigate the effect of porosity and binder hydrophobicity on the redox targeting reaction between LFP/FePO_4_ and [Fe­(CN)_6_]^4–/3–^. We determine the conversion rate of the booster via *in situ* UV–Vis spectroscopy on the posolyte and assess the rate-dependent capacity utilization and booster durability in symmetric cell testing under realistic flow conditions. We show that the replacement of PVDF with a non-fluorinated binder such as polycaprolactone (PCL) or cellulose acetate (CA) results in a higher conversion rate and improved capacity utilization in LFP booster pellets without compromising booster stability. Our results demonstrate that binder hydrophobicity governs electrolyte penetration in the booster composite, highlighting its crucial role for RTFB performance.

Pellets with different formulations were produced via solvent-assisted extrusion. A mixture of carbon-coated LFP powder (1.5 wt% carbon) and binder solution was extruded to form pellets of 1 mm diameter and approximately 5 to 15 mm length (Figure S1). An example of the as-obtained samples, denoted as *non-porous*, is shown in Figure 1a. Porosity was introduced by adding potassium chloride (KCl) to the extrusion mixture as a sacrificial pore-former and subsequently removing it by washing the pellets in water (Figure S2). The resulting samples are referred to as *porous*, an example of which is depicted in Figure 1b showing the macropores introduced by KCl. Porous and non-porous pellets were formulated with PVDF or PCL, with the binder content set to a comparatively low value of 5 wt% in all cases to minimize the fraction of inactive components. The pellets are mechanically stable and easy to handle after drying. The PCL pellets resist deformation under loads of up to 870 g, exceeding reported mechanical strength of comparable boosters (Figure S3).^36^ Porous boosters are deformed more easily, but PVDF and PCL samples form cohesive pellets even after pressing at 200 kg (Figure S4). Larger fractions of binder are not investigated here, and in the interest of maximizing energy density, smaller percentages should be considered in future work.

**Figure 1:**
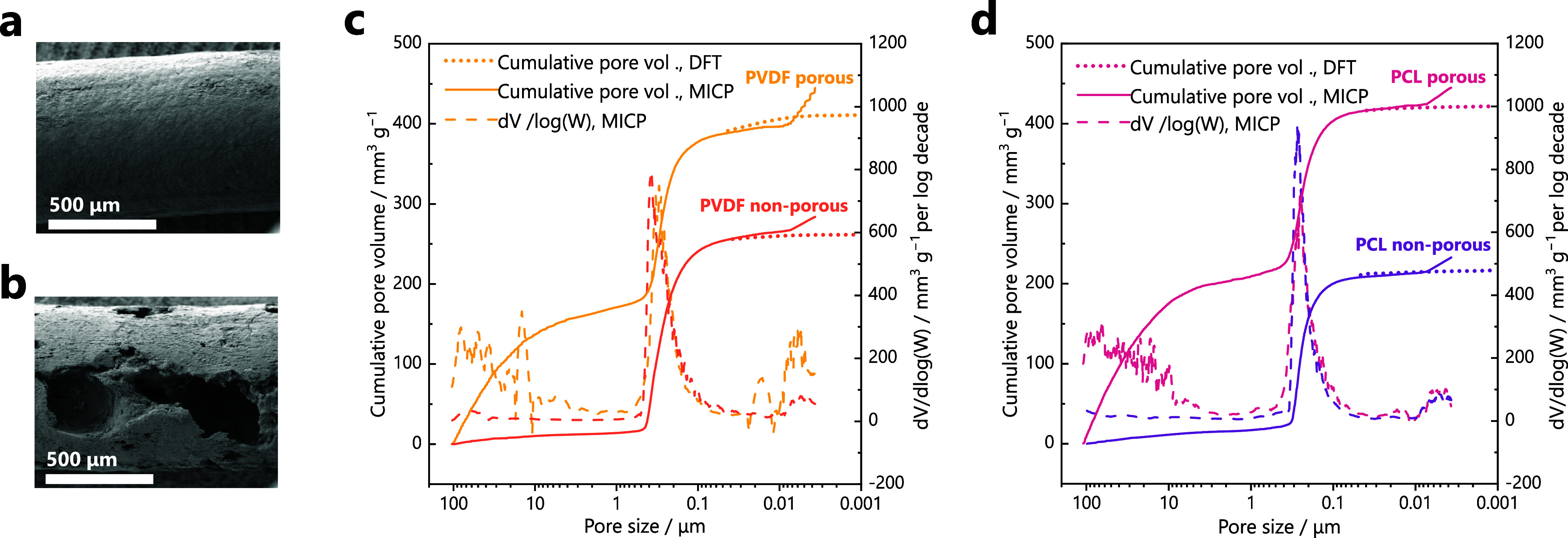
Scanning electron microscopy images of (a) non-porous and (b) porous LFP pellets with 5 wt% binder content. (c-d) Pore size distributions of pellets formulated with PVDF and PCL, respectively. Dotted lines correspond to the cumulative pore volume from nitrogen adsorption experiments (DFT kernel). The solid lines correspond to the cumulative pore volume and the dashed lines to the pore size distribution obtained from mercury intrusion capillary pressure (MICP) porosimetry experiments.

PCL is a non-fluorinated, biodegradable polyester commonly found in biomedical applications.^37^ Like PVDF, it is insoluble in water and moderately soluble in *N,N*-Dimethylformamide (DMF), which was used as a solvent to create the binder solutions in this work. Note that KCl was selected as pore-former over LiCl because the latter is soluble in DMF, resulting in mechanically unstable pellets. The porosity generated by the KCl templating was consistent across PVDF and PCL pellets, with a total pore volume increase of approximately 0.2 cm^3^ g^–1^ in porous samples compared to non-porous ones (Figure 1c-d). The treatment exclusively created macropores larger than ∼1 μm, with a substantial fraction in the 10–100 μm range, while the pore size distribution below 1 μm remained unchanged. The pellets showed only a minor contribution of pores <50 nm to both total porosity and specific surface area. The BET surface area of PCL and PVDF pellets was relatively low (<10 m^2^ g^–1^) and largely unaffected by the KCl treatment (Table S1). Thus, differences in booster performance are not governed by specific surface area.

To assess the reaction rate between the solid booster pellets and the redox mediator, *in situ* UV–Vis spectroscopy measurements were performed. The approach is based on the decrease of absorbance at 420 nm upon reduction of [Fe­(CN)_6_]^3–^ to [Fe­(CN)_6_]^4–^ in contact with LFP, from which the amount of LFP oxidized to FePO_4_ per unit time can be calculated (Figure S5).^20,23^ We explored two different modes of operation for the UV–Vis measurement: The static mode represents the commonly described approach, where boosters are placed directly in the electrolyte tank. As shown schematically in Figure 2a, the LFP pellets were placed into an aqueous [Fe­(CN)_6_]^3–^ solution which was continuously sampled through a flow cuvette in the UV–Vis spectrometer by a peristaltic pump. From the absorbance spectra, the amount of LFP converted over time was calculated. The results shown in Figure 2b indicate a higher conversion rate for the porous pellet formulations over the non-porous ones: While the conversion reaches 20% and 22% after 1 h for porous PVDF and PCL pellets, respectively, only 14% and 13% are achieved with the non-porous pellets. For comparison, Lotenberg et al. reported LFP conversions over 1 h of <5% for non-porous and around 20% for porous pellets containing 50 wt% PVDF.^24^

**Figure 2:**
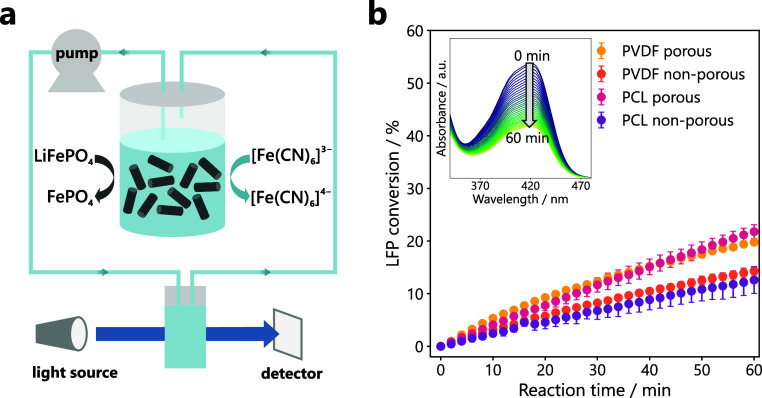
(a) Schematic of the experimental setup used for the *in situ* UV–Vis experiments in static mode, with LFP pellets placed directly into the electrolyte tank and continuous sampling of the electrolyte to the flow cuvette. (b) Percentage of reacted LFP in different pellet formulations determined via UV–Vis measurement over 1 h. 50 mL of 20 mM K_3_[Fe­(CN)_6_] was used as electrolyte to achieve a 1:1 molar ratio of LFP:[Fe­(CN)_6_]^3–^. Measurements were performed in triplicate for each pellet formulation; standard deviations are shown as error bars. The inset depicts an exemplary absorbance spectrum showing the decrease in absorbance at 420 nm over 1 h.

The second mode of operation, shown schematically in Figure 3a, is designed to overcome mass transport limitations in the static setup by forcing the electrolyte flow through a reactor containing the LFP pellets. The reactor was resin 3D printed, and dimensions are provided in the Supporting Information (Figure S6). In this flow mode, the pellets formulated with PCL reach significantly higher conversion rates compared to the PVDF pellets. As shown in Figure 3c, the conversion of LFP reaches 55% and 38% over 1 h with porous and non-porous PCL pellets, respectively, while around 20% is achieved with both PVDF formulations. LFP conversion more than doubled compared to the static mode with PCL pellets, while only a small difference was observed in the case of PVDF. Furthermore, large porosity enhances the conversion in PCL pellets considerably, while little benefit is observed with PVDF. Even in flow mode, the pores in PVDF pellets are evidently inaccessible to the liquid electrolyte resulting in low conversion rates. Importantly, the error obtained with the porous PVDF pellets is much larger compared to its non-porous counterpart. Slow release of air bubbles from the porous PVDF pellets was observed in the static setup, whereas porous PCL pellets did not release air. Therefore, we suspect that in flow mode, air bubbles are trapped within porous PVDF pellets.

We hypothesize that the higher LFP conversion rates observed for PCL formulations can be explained by the lower hydrophobicity of PCL compared to PVDF. To confirm this conjecture, water contact angle analyses were conducted on flat, slurry-cast LFP electrodes with 5 wt% binder content, the same amount as for the pellets. As shown in Figure 3c, the contact angle of 135° on the LFP-PVDF composite is much higher compared to the angle of 89° on LFP-PCL, even at this relatively low binder concentration. PVDF is known for its strong hydrophobicity caused by the lack of functional groups with hydrogen bonding capability. While PCL is also categorized as hydrophobic,^37,38^ its ester functionality can form hydrogen bonds with water and it is non-fluorinated, resulting in a much lower water contact angle. Given that the electrolyte wettability increases with smaller contact angles, the higher LFP conversion rate in PCL pellets can be explained by improved electrolyte penetration and increased accessible surface area. Indeed, LFP-PCL composites showed a higher capacitance compared to LFP-PVDF, indicating that more surface area is accessible to the aqueous electrolyte in the presence of the less hydrophobic binder (Figure S7). The more hydrophobic surface of PVDF pellets also explains why air bubbles are trapped more easily inside pores compared to PCL pellets. This is consistent with experiments, where a pressure of 70 bar was insufficient to push water into 7 nm hydrophobic pores.^39^

The difference in wettability between PCL and PVDF composites seemingly contradicts the results obtained in the static UV–Vis setup (Figure 2), which show a similar conversion rate for PCL and PVDF pellets. However, with only minimal electrolyte recirculation to the UV–Vis in this setup, transport around the pellets is effectively film-limited, as a thick external concentration boundary layer forms and controls the overall reaction rate. Under this condition, the geometry of the pellets is crucial for the performance: micron-scale through-pores (1–100 μm) give porous pellets more developed external area and shorter internal diffusion paths, leading to increased conversion rates over non-porous pellets. Minor background mixing from the peristaltic pump primarily thins the external film without altering internal transport. Consequently, conversion in the static setup is plausibly dominated by pellet geometry rather than electrolyte wetting, explaining why porous PCL and PVDF pellets perform similarly and outperform non-porous pellets. Introducing forced flow (Figure 3) collapses the external film resistance for mass transport, shifting control inside the pellets. We hypothesize that increased hydrophilicity of PCL pellets enables more continuous aqueous pathways, which allow the micron-scale pores to act as through-channels. This enables intraparticle advection, analogous to perfusion chromatography where micron-size through pores accelerate internal transport.^40^ In contrast, hydrophobic pore walls resist water entry. Because of the clogging of the pores with air bubbles, the permeability collapses and the liquid electrolyte then exists mainly as thin wetting films along the walls. Diffusion in such nanometric films is orders of magnitude slower than in bulk water.^41^ Therefore, pellets formulated with PVDF remain diffusion-limited inside and insensitive to bulk flow, so the conversion rates of porous and non-porous PVDF pellets are similar in the flow and the static setup, while the more hydrophilic PCL pellets enables significantly higher conversion in the flow mode.

To further investigate the relationship between binder hydrophobicity and conversion rate, additional pellets with cellulose acetate (CA) as a binder were also prepared. CA is a biodegradable polymer derived from cellulose and offers variable hydrophobicity depending on the degree of substitution with acetyl groups. Here, the partially substituted cellulose diacetate is used. Like PCL and PVDF, it is insoluble in water but soluble in DMF, which enables an analogous booster extrusion procedure. Only porous CA pellets were investigated here, as the performance of non-porous pellets was shown to be inferior in the case of PCL, and the behavior of CA formulations was expected to be similar. Indeed, although the CA pellets were more brittle (Figure S3), an almost identical LFP conversion of 53% over 1 h is observed for the pellets formulated with CA, compared to the previously discussed 55% in the case of porous PCL pellets (Figure S8). The water contact angle of 84° measured on CA containing electrodes is comparable to the value determined for PCL and supports the hypothesis that lower binder hydrophobicity leads to higher conversion rates in the present booster–mediator system.

**Figure 3:**
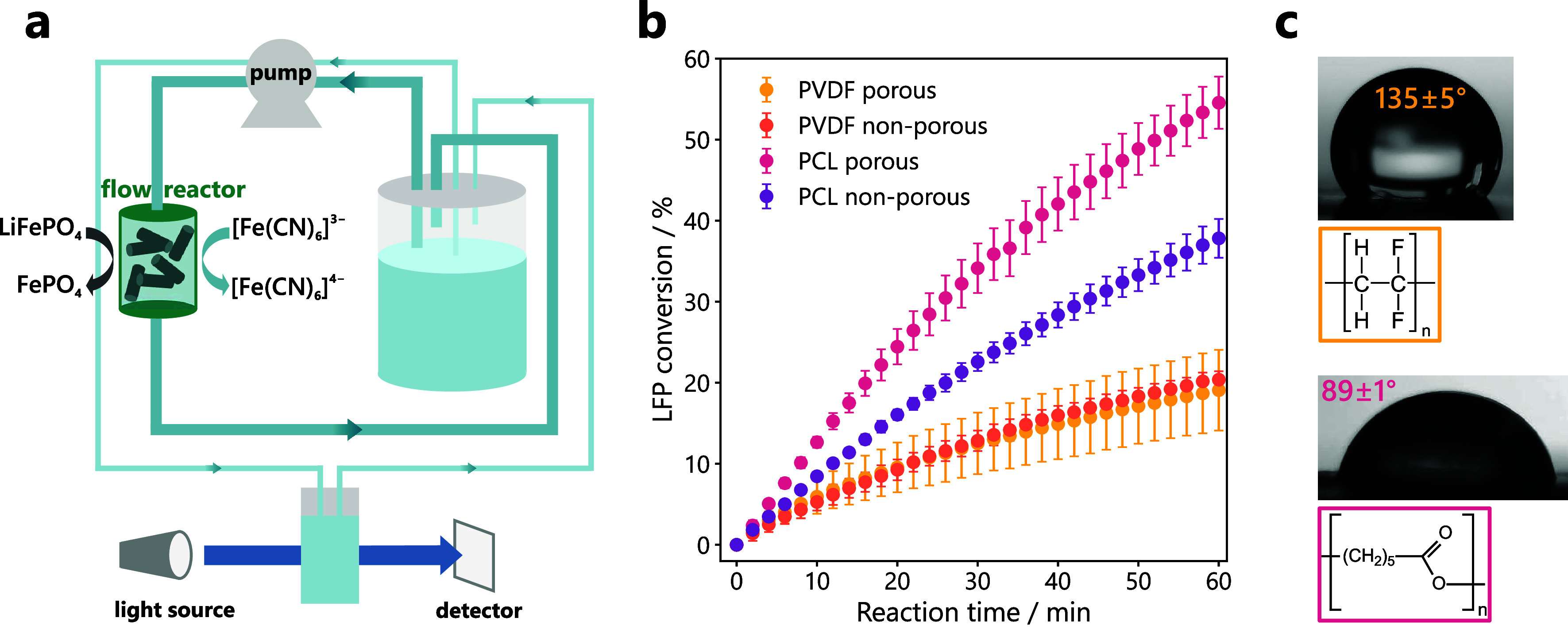
(a) Schematic of the experimental setup used for the *in situ* UV–Vis experiments in flow mode, with LFP pellets contained in a 3D-printed flow reactor, which is placed in the electrolyte stream. The electrolyte is sampled continuously in a separate liquid loop. (b) Percentage of reacted LFP in different pellet formulations determined via flow UV–Vis measurement over 1 h. 50 mL of 20 mM K_3_[Fe­(CN)_6_] was used as electrolyte to achieve a 1:1 molar ratio of LFP:[Fe­(CN)_6_]^3–^. Measurements were performed at least in triplicate for each pellet formulation; standard deviations are shown as error bars. (c) Water droplets on LFP–binder composite electrodes (95:5 ratio of LFP:binder) with the corresponding average contact angle values with standard deviations and chemical structures for polyvinylidene fluoride (yellow) and polycaprolactone (pink). Measurements were performed in triplicate for each electrode composition.

The influence of the binder on the LFP conversion rate was further investigated with rate tests in symmetric flow batteries^42^ with 25 mL of 0.1 M [Fe­(CN)_6_]^3–^ as negolyte and 10 mL of 0.1 M [Fe­(CN)_6_]^4–^ as posolyte, both in 0.5 M LiCl and 20 vol% dimethyl sulfoxide (DMSO). 20 vol% of DMSO was added to the negolyte and the posolyte to match the oxidation potential of [Fe­(CN)_6_]^4–^ to the reduction potential of FePO_4_.^23^ The rate tests consisted of galvanostatic charge–discharge cycling at different current densities, with five cycles being completed in each step. First, the baseline capacity of the capacity-limiting posolyte was established without any boosters, with a cycling step at 20 mA cm^–2^, of which an exemplary voltage profile is shown in Figure 4a. The theoretical electrolyte capacity of 26.8 mAh was not reached experimentally, possibly due to small liquid losses during transfer and/or kinetic limitations. For the rate test, LFP booster pellets were then placed into the flow reactor which was attached to the electrolyte outlet on the positive side of the flow cell. This reactor placement enables the contact between the booster and the charged mediator species flowing out of the cell before the latter gets diluted in the electrolyte tank, which adds to the driving force of the reaction.^23,31^ Voltage profiles of each galvanostatic step of the rate test are shown in Figure S9.

The theoretical capacity of the battery after the addition of one molar equivalent of LFP is 53.8 mAh, double the theoretical capacity of the capacity limiting posolyte. At the lowest current density of 1 mA cm^–2^, the measured charge capacity almost reaches the theoretical maximum when pellets formulated with CA or PCL were used, as shown in Figure 4b. The CA and PCL pellets enable a higher capacity than PVDF formulations at 2 and 5 mA cm^–2^ as well. However, no distinct difference in the capacity can be observed at current densities above 10 mA cm^–2^. At these higher rates, the booster has little time to react with the mediator and contribute capacity, as each half-cycle takes only 15–30 min. To allow for a more meaningful comparison, the capacity utilization was calculated as the ratio of the experimental and theoretical charge capacity of the solid booster alone. The results, presented in Figure 4c, show a capacity utilization of close to 100% for CA and PCL pellets at 1 mA cm^–2^, while PVDF pellets reached around 85% at the same rate. For comparison, Vivo-Vilches et al. reported 45% capacity utilization with porous LFP pellets at this rate,^23^ showing that booster utilization is improved significantly by the flow reactor we propose here, even with a hydrophobic binder. In general, booster utilization was lower when PVDF was used as a binder, except at 15 and 20 mA cm^–2^, where again no distinct difference was observed between the different pellets. A decline in capacity utilization with increasing current density was observed for every pellet formulation, suggesting that the reaction rate is limited by factors other than surface accessibility. For example, large LFP particles could hinder Li^+^ and electron transport, or preferential flow channels could induce mass transport limitations.^22^ The difference in performance between PCL/CA and PVDF was less pronounced than in the UV–Vis measurements, which can be attributed to the longer time scales of the symmetric cell tests. While the UV–Vis experiments capture LFP conversion within the first hour of electrolyte contact, the first step of the rate test alone lasted around 50 h, long enough for wetting effects to equilibrate. Additionally, the presence of DMSO in the electrolyte decreases its hydrophilicity, improving relative wettability of the PVDF pellets. As shown in Figure S10, the contact angle of the electrolyte is lower compared to pure water on PVDF composites. Despite these effects, the use of less hydrophobic binders such as PCL and CA consistently enhances booster utilization.

**Figure 4:**
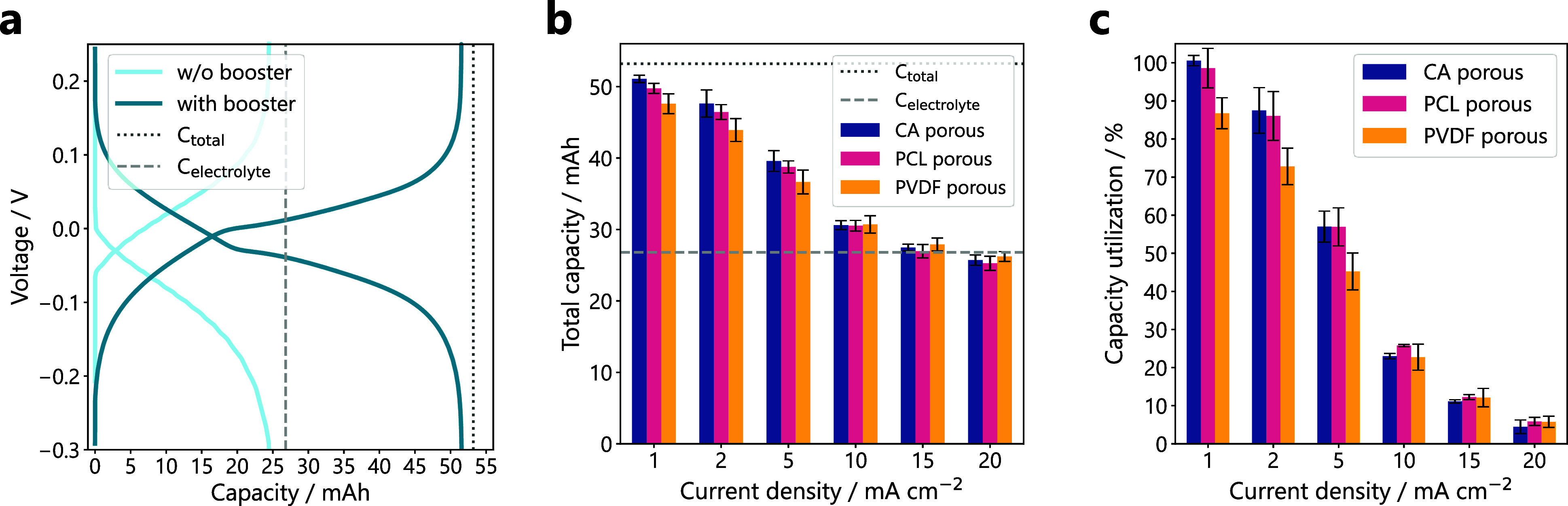
(a) Example of a galvanostatic voltage profile of a symmetric flow battery with 25 mL of 0.1 M [Fe­(CN)_6_]^3–^ as negolyte and 10 mL of 0.1 M [Fe­(CN)_6_]^4–^ as posolyte, both in 0.5 M LiCl and 20 vol% DMSO. To establish the baseline capacity of the electrolyte, a galvanostatic cycling step was performed at 20 mA cm^–2^ before addition of booster pellets containing one molar equivalent of LFP and cycling at 1 mA cm^–2^. The dashed line represents the theoretical capacity C_electrolyte_ of the posolyte (capacity-limiting side) and the dotted line represents the theoretical capacity C_total_ of both the posolyte and the solid boosters together. (b) Average total charge capacity and (c) average booster capacity utilization achieved for different pellet formulations in the symmetric flow battery during galvanostatic cycling at different current densities. Five cycles were performed at each current density and the charge capacity of the 5th cycle was used for the calculations. Measurements were performed in triplicate for each pellet formulation; standard deviations are shown as error bars.

Dried booster pellets before and after the rate tests show no structural changes (Figure S11). To further assess the durability of PVDF-alternatives, the boosters were submerged in ethanol for six months. Ethanol interacts more strongly with CA and PCL than water and can induce swelling in PVDF-based materials,^43^ representing a more aggressive solvent environment than an aqueous electrolyte. Yet, no pellet disintegration or loss of mechanical integrity was observed (Figure S12). Together with the absence of mechanical or morphological degradation after symmetric cell cycling under flow, these results indicate that replacing PVDF does not compromise long-term stability of the booster composites.

Replacing PVDF with non-fluorinated binders in solid booster composites offers a simple, immediately applicable strategy to improve both the performance and sustainability of aqueous RTFBs. LFP conversion rate increases by almost three times by employing PCL or CA, two representative non-fluorinated binders. The improved wettability provided by these relatively hydrophilic materials facilitates penetration of the aqueous electrolyte into the pellets, thereby accelerating the reaction of the booster with the redox mediator. Introducing porosity further increased conversion rates in PCL pellets while no positive impact was observed with PVDF, indicating that pores are insufficiently accessible to the electrolyte in PVDF formulations. Hence, our work highlights mass transport limitations inherent to liquid-phase operation with pelletized boosters. Both external and internal liquid transport can be optimized, for example by forced convection to thin boundary layers and by incorporating through-pores that promote convective transport. Non-fluorinated LFP boosters also exhibited improved capacity utilization during charge–discharge cycling in symmetric flow cells, with both CA and PCL reaching nearly 100% at 1 mA cm^–2^. Given the simplicity and effectiveness of this modification, the replacement of PVDF with non-fluorinated, less hydrophobic binders should be readily adopted across aqueous RTFB systems.

## Supplementary Material



## Data Availability

The data supporting the findings of this study are openly available in Zenodo at 10.5281/zenodo.17571171.

## Notes

Details on experimental procedures, pellet preparation, MICP and BET measurements, UV–Vis spectroscopy, capacitance measurements, galvanostatic voltage profiles, and contact angle analysis (PDF)
