# Supplementary material for: Enhancing Solid Booster Utilization in Redox-targeted Flow Batteries with Non-fluorinated Binders
Source: ACS Mater Lett. 2026 Feb 3;8(3):889–95. doi: 10.1021/acsmaterialslett.5c01591 (PMC12958333; doi:10.1021/acsmaterialslett.5c01591)
Supplement: Supplementary file 1 [file tz5c01591_si_001.pdf]

Supporting Information for

Enhancing Solid Booster Utilization in Redox-targeted Flow Batteries with Non-fluorinated Binders

Julia Lorenzetti,[1,2] Paweł P. Ziemiański,[1] Cédric Kupferschmid,[1] David Reber,[1,*]

1) Empa, Swiss Federal Laboratories for Materials Science and Technology, 8600 Dübendorf, Switzerland

2) École Polytechnique Fédérale de Lausanne, Institute of Materials, 1015 Lausanne, Switzerland

*E-mail: david.reber@empa.ch

Materials

All reagents and solvents were used as received. Potassium ferrocyanide trihydrate (>99%), potassium ferricyanide (>99%), dimethyl sulfoxide (≥99%) and polycaprolactone (M.W. approx. 80'000) were purchased from Thermo Scientific. *N,N*-Dimethylformamide (99.8%), lithium chloride (99%) and cellulose acetate (average M.W. 30'000, 39.8 wt% acetyl) were purchased from Sigma Aldrich. Lithium iron phosphate (1.5 wt% carbon coating) and polyvinylidene difluoride (>99.5%, M.W. 1'000'000) were purchased from AME Energy Co., Limited. Potassium chloride (99%) was purchased from Alfa Aesar. Aqueous solutions were prepared using ultrapure water (Milli-Q EQ 7000, 18.2 MΩ cm at 25 °C).

Pellet preparation

To prepare the booster pellets, LFP powder and a binder solution (and KCl for porous pellets) were first combined and homogenized by hand until no loose powder was left. Then, the mixture was extruded with a Biomation CML Multilab Extruder/Granulator at 100 rpm using 1 mm diameter dyes. The resulting pellets of 1 mm diameter and ~5–10 mm length were dried in a vacuum oven at 60 °C overnight. Pellets prepared with KCl as pore-former were then stirred in ~100 mL of ultrapure water for 1 hour and stirred again overnight after exchanging the water to remove KCl, leaving the pellets with a porous structure. For each of the three binders, a slightly different formulation was used to prepare the extrusion mixture to account for relative viscosities of binder solutions: *PVDF*: A 15 wt% solution of PVDF in DMF was prepared by stirring 1.5 g of PVDF in 8.5 g of DMF at 50 °C overnight. The extrusion mixture consisted of 9.5 g of LFP and 3.33 g of the 15 wt% binder solution to achieve a final binder content of 5 wt%. *PCL*: A 18.5 wt% solution of PCL in DMF was prepared by stirring 1.85 g of PCL in 8.15 g of DMF at 50 °C overnight. The extrusion mixture consisted of 9.5 g of LFP and 2.70 g of the 18.5 wt% binder solution to achieve a final binder content of 5 wt%. *CA*: A 19.5 wt% solution of CA in DMF was prepared by stirring 1.95 g of CA in 8.05 g of DMF at 50 °C overnight. The extrusion mixture consisted of 9.5 g of LFP and 2.56 g of the 19.5 wt% binder solution to achieve a final binder content of 5 wt%. For each of the three binders, the porous pellets were produced by adding 6 g of KCl to the extrusion mixture. Considering the carbon coating of the LFP powder used, the final pellet composition is 93.75:1.25:5 (LFP:carbon:binder).

Physical characterization

Scanning electron microscopy (SEM)

SEM images were recorded on a ThermoFisher Scientific Phenom XL G using the secondary electron beam detector and an accelerating voltage of 5 kV. The pellets were directly attached to a sample holder with sticky carbon tape before inserting them into the vacuum chamber, which was then evacuated to 0.1 Pa.

Mercury intrusion capillary pressure porosimetry (MICP)

MICP measurements were performed with a porosimeter consisting of a Low Pressure Pascal 140 and a High Pressure Pascal 440 unit by ThermoFisher Scientific. The pressure was increased by 6–19 MPa min^{-1} with a maximum pressure of 400 MPa. The surface tension of mercury was set to 0.48 N m^{-1} and a contact angle of 140° was used.

Characterization of microtexture by nitrogen adsorption

Subcritical nitrogen adsorption–desorption isotherms were collected on a 3Flex analyzer (Micromeritics). For each measurement between 1 and 4 g of the sample was loaded into glass sample tubes and activated under high vacuum (~1 × 10^{-3} mbar) at 100 °C for 24 h. Isotherms were recorded at 77.3 K (liquid N_2 bath) over p/p_0 = 0.001–0.998. The Brunauer–Emmett–Teller (BET) specific surface area and pore size distributions were obtained with the Micromeritics Flex 6.03 software. The BET specific surface was calculated using Rouquerol criteria and N_2 cross-section of 0.162 nm^2. Pore size distributions in the micro/mesopore (~1–50 nm) range were calculated using non-local density functional theory (NLDFT) with the HS-2D-NLDFT (Carbon, N_2, 77 K) kernel as implemented in the Micromeritics software. Introduction of porosity by salt-templating did not significantly modify specific surface areas of the samples, as shown in Table S1. Cumulative pore volumes from NLDFT were shifted to match the MICP cumulative pore volumes for the pores of ~50 nm. The significant increase in the pore volume in MICP distribution recorded for pores ~10 nm is an artefact, due to either sample compressibility (compression of blocked pores, or compression of the sample matrix due to high-pressure), or filling of the much bigger pores (>10 nm) through the pore throats in that size range.

Table S1. Specific surface area BET for the different pellet formulations.

	**PVDF non-porous**	**PVDF porous**	**PCL non-porous**	**PCL porous**	**CA porous**
**BET surface area (m^2 g^{-1})**	1.4	5.0	2.7	2.7	5.2

UV–Vis spectroscopy

The UV–Vis spectroscopy measurements were performed on a ALS SEC2020 UV–Vis Spectrometer which was referenced with water prior to every measurement series. A quartz flow cuvette with 0.1 cm path length from Starna Scientific Ltd. was used to measure a 2 mM solution of ferricyanide, consisting of 1 mmol (329.4 mg) of $K_3Fe(CN)_6$ dissolved in 50 mL of water. The solution was pumped through the flow cuvette at 50 mL min^{-1}, using a Masterflex pump and Easy-Load II pump head, and its absorption measured as background before adding 166 mg of pellets (~1 mmol of LFP) either directly to the tank or to the flow reactor and circulating the solution over the pellets. Absorption spectra were then measured every 2 min for 60 min total. This measurement was performed at least three times for every pellet formulation.

Conversion calculation: Because the reactant $[Fe(CN)_6]^{3-}$ has a strong absorbance peak around 420 nm and the product $[Fe(CN)_6]^{4-}$ has no absorbance in this region, the intensity of the measured UV–Vis spectra at 420 nm can serve as a measure of the amount of converted $[Fe(CN)_6]^{3-}$. With a 1:1 molar ratio of LFP:$[Fe(CN)_6]^{3-}$, the conversion of LFP to $FePO_4$ can also be calculated from the decrease in absorbance at 420 nm over time.

$$\text{LFP conversion (\%)} = (1 - \frac{A_{420}(t)}{A_{420}(0)}) * 100$$

The flow reactor with an inner volume of approximately 1 mL was 3D-printed with a Creality Halot Mage Pro 8k printer using Liqcreate Strong X water washable resin (Figure S6). It includes a net at the outlet to prevent the booster pellets from exiting the reactor and blocking the tubing.

Contact angle

The water contact angle was measured on slurry cast electrodes with the same composition as the booster pellets. They were prepared by combining 1 g of a 5 wt% binder solution of either PVDF, PCL or CA in DMF and 0.95 g of LPF and homogenizing this mixture in a Thinky ARE-250 slurry mixer at 2000 rpm for 20 min. From this slurry, thin films of 100 µm thickness were cast onto titanium foil with a Sheen automatic Film Applicator, which were dried at 80 °C overnight. From the films, electrodes of 11 mm diameter were punched out and dried under vacuum at 60 °C overnight. The measurements were carried out on a Ossila L2004 contact angle goniometer and the included Ossila software was used for analysis. Videos of 10 min length were recorded at 1 frame per second on three different electrodes for every binder.

Flow cell setup

Fuel cell type flow cells with gold coated current collectors and serpentine graphite flow fields were purchased from Fuel Cell Technologies Inc. Fumatech E-630(K) membranes were soaked in ultrapure water before use. Two stacked sheets of non-activated GDL 39 AA carbon paper (SGL, 280 µm thick, 5 cm2 each) were used on either side of the membrane with 1/64-inch Viton gaskets providing ~25% electrode compression. The cells were tightened with a torque wrench set to 6 N m. The electrolytes were circulated at a rate of 60 $mL\,min^{-1}$ through LS16 Norprene tubing with a peristaltic pump (either Watson-Marlow 323S with a 313D pumphead or Longer WT600-2J). The following electrolyte compositions were used for the symmetric cell rate tests: *Negolyte*: 25 mL of 0.1 M $K_3Fe(CN)_6$ and 0.5 M LiCl in a 20:80 DMSO:water mixture; *posolyte* (capacity limiting side): 10 mL of 0.1 M $K_4Fe(CN)_6$ (corresponds to 1 mmol) and 0.5 M LiCl in a 20:80 DMSO:water mixture.

Electrochemical characterization

Electrochemical measurements on symmetric flow cells were recorded on a Biologic potentiostat (either SP-150e or SP-240). The protocol of the performed rate tests consisted of a galvanostatic charge/discharge at 20 $mA\,cm^{-2}$ to determine the baseline charge capacity provided by the electrolyte alone. After the addition of 166 mg of booster pellets (≈1 molar equivalent of LFP) to the flow reactor, six galvanostatic charge/discharge steps of increasing current density (±1, 2, 5, 10, 15, and 20 $mA\,cm^{-2}$) within a voltage window of −0.3 to +0.3 V were performed. Five cycles were recorded for each galvanostatic step.

The capacity utilization was calculated as follows:

$$\text{capacity utilization (\%)} = \frac{(C_{tot} - C_{electrolyte})}{C_{theo}} * 100$$

where C_{tot} is the charge capacity of booster and electrolyte combined, $C_{electrolyte}$ is the charge capacity of the electrolyte at 20 $mA\,cm^{-2}$, and C_{theo} is the maximum theoretical capacity of the added booster pellets, assuming a capacity of 170 $mAh\,g^{-1}$ for LFP. The charge capacity in the 5th cycle of each rate test step was used for the calculation.

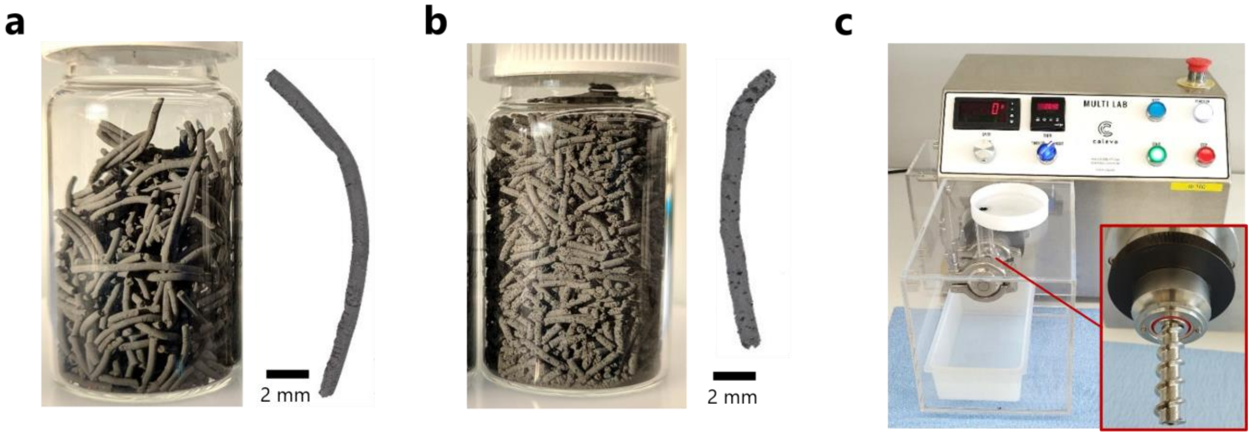

Figure S1. Images of **(a)** non-porous and **(b)** porous LFP pellets produced with the **(c)** Biomation CML Multilab Extruder/Granulator.

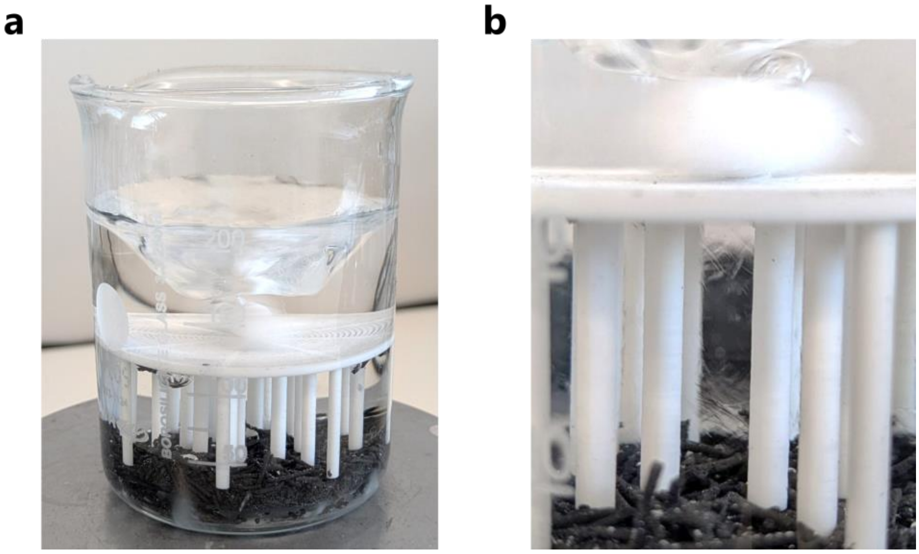

Figure S2. (a) Experimental setup for removing KCl from the pellets by washing them in ultrapure water, including a 3D-printed net to protect the pellets from contact with the magnetic stirrer. **(b)** Schlieren caused by salt crystals being removed from the pellets.

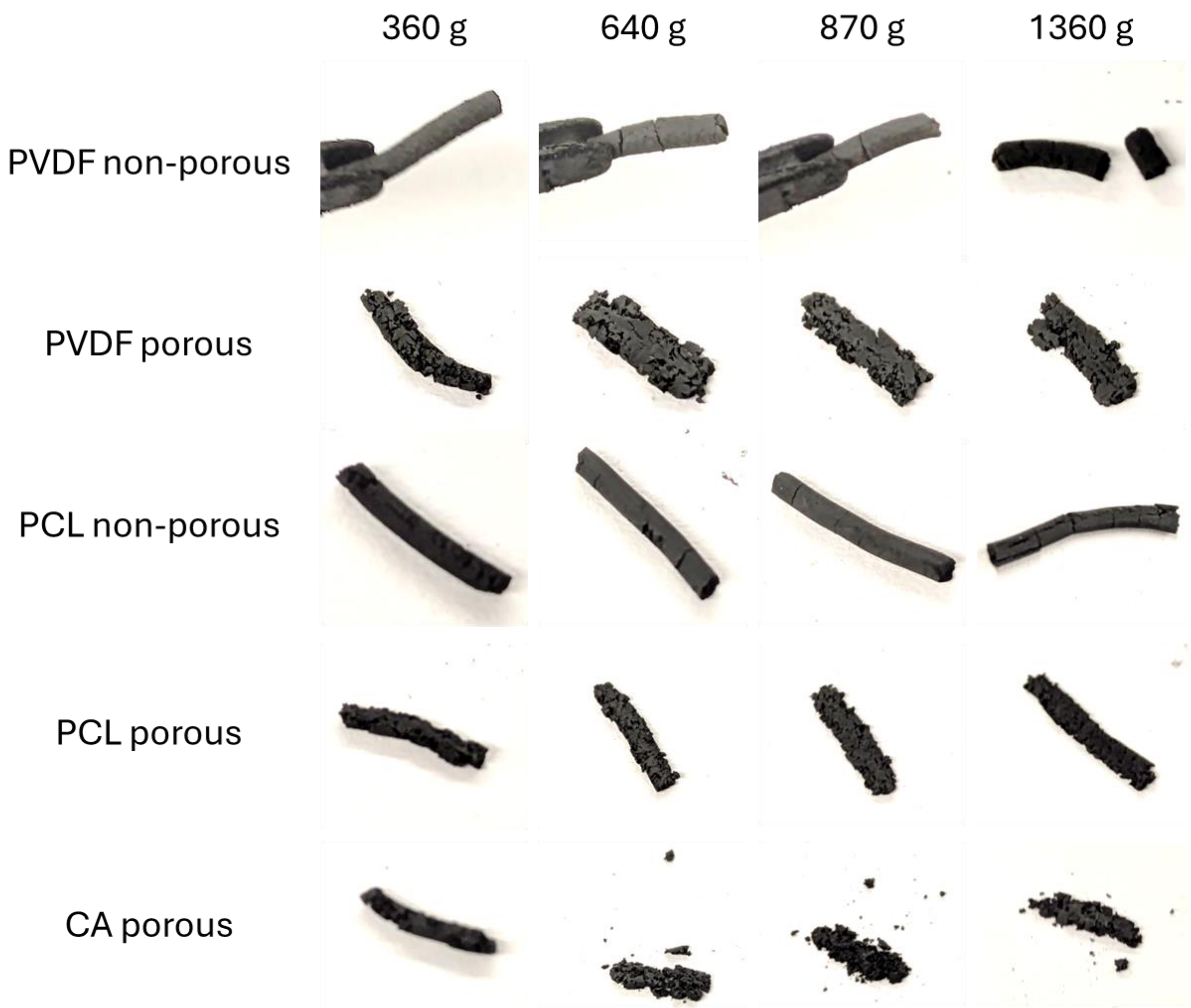

Figure S3. Mechanical strength test of boosters. Individual pellets with ca. 5 mm length were placed centrally under steel cylinders weighing 360, 640, 870, or 1360 g for one minute. Porous pellets are deformed more easily. PVDF and PCL pellets are pliable and do not disintegrate upon deformation, while CA pellets are brittle and crushed above 360 g.

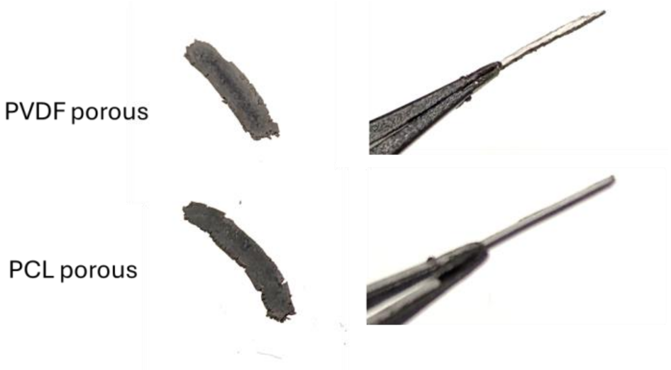

Figure S4. Booster pellets after uniaxial pressing at 200 kg for one minute.

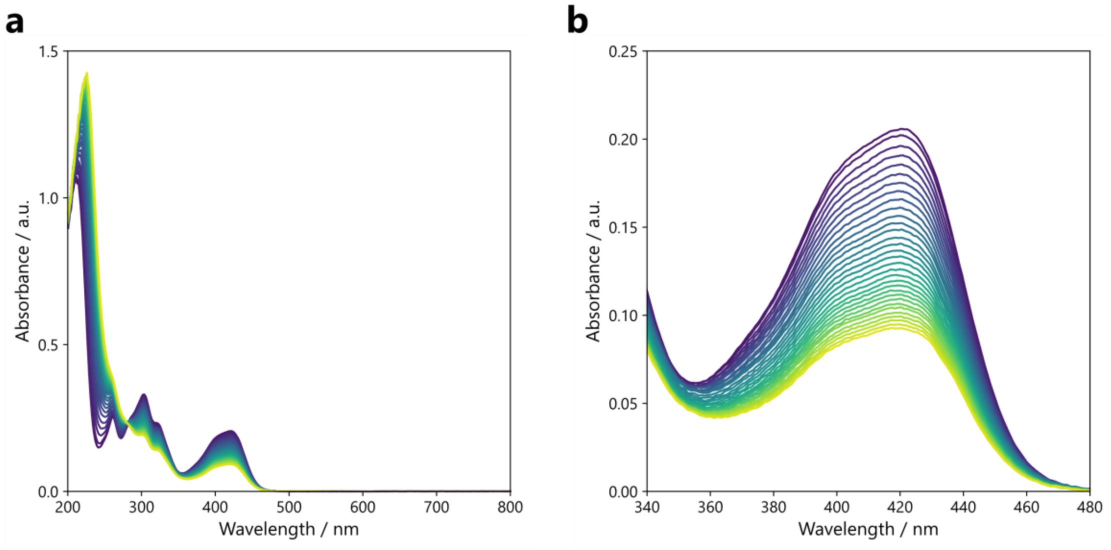

Figure S5. (a) full and **(b)** zoomed-in UV–Vis spectrum of a $[Fe(CN)_6]^{3-}$ solution measured in flow mode over one hour in the presence of 1 molar equivalent of porous LFP pellets containing PCL as binder.

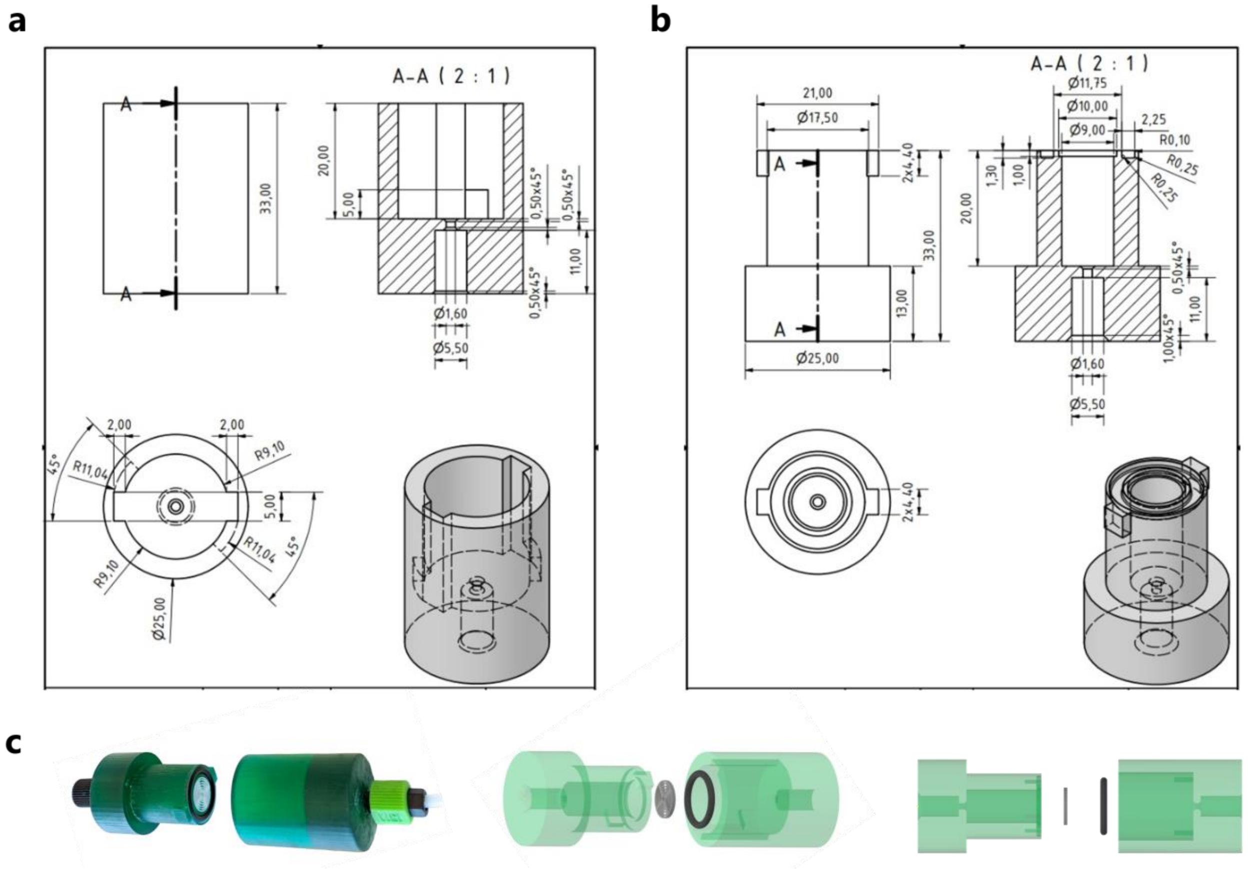

Figure S6. Technical drawings of the **(a)** female and **(b)** male part of the flow reactor. **(c)** Image and digital visualizations of the flow reactor, including a net and O-ring.

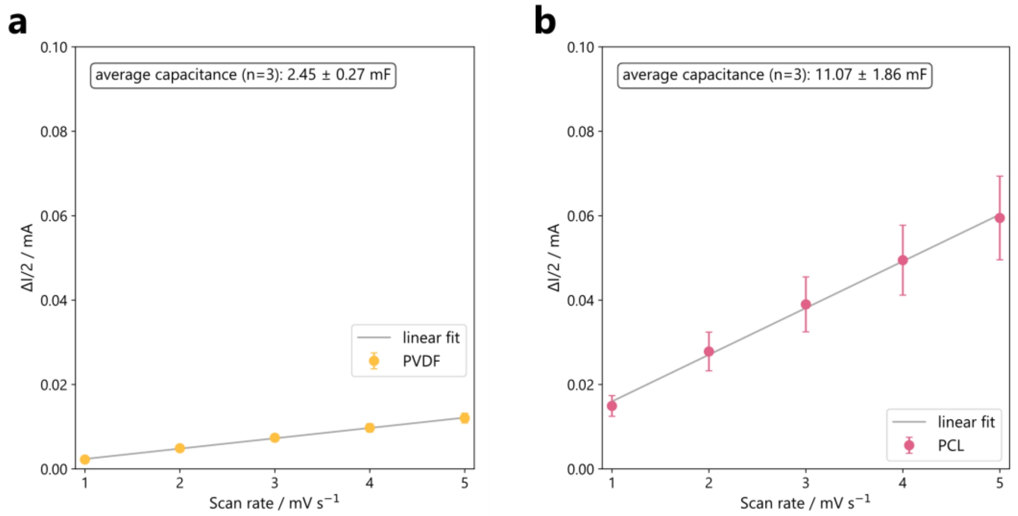

Figure S7. Average capacitance as determined by cyclic voltammetry for **(a)** LFP-PVDF and **(b)** LFP-PCL composite electrodes (95:5 LFP:binder). Measurements were performed in a Swagelok cell with the LFP:binder composite as the working electrode, activated carbon as the counter electrode and Ag/AgCl$_{3M\ NaCl}$ as the reference electrode. 250 µL of 0.5 M LiCl served as the electrolyte. CV scans were performed between −0.05 V and +0.05 V vs. Ag/AgCl and the current at 0 V vs. Ag/AgCl was used to determine the capacitance. Measurements were performed in triplicate; the standard deviations are shown as error bars.

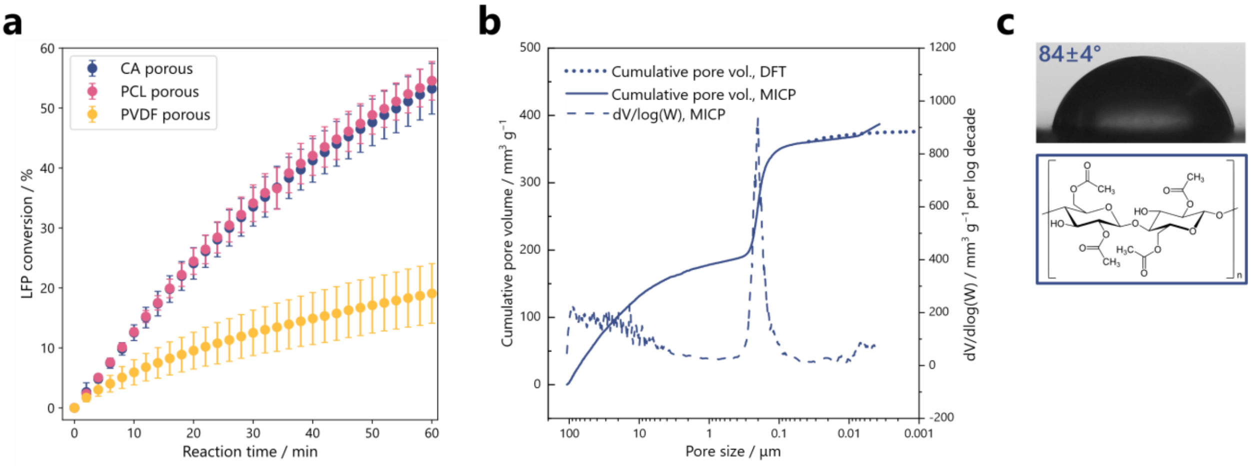

Figure S8. (a) Percentage of reacted LFP with different pellet formulations determined by flow UV–Vis measurement over one hour. 50 mL of 20 mM $K_3[Fe(CN)_6]$ was used as electrolyte to achieve a 1:1 molar ratio of LFP:$[Fe(CN)_6]^{3-}$. Measurements were performed in triplicate for each pellet formulation; standard deviations are shown as error bars. **(b)** Pore size distributions of porous pellets formulated with CA. Dotted lines correspond to the cumulative pore volume from nitrogen adsorption experiments (DFT kernel). The solid lines correspond to the cumulative pore volume and the dashed lines to the pore size distribution obtained from mercury intrusion capillary pressure (MICP) porosimetry experiments. **(c)** Water droplet on an LFP-CA composite electrode (95:5 ratio of LFP:CA) with the corresponding average contact angle values with standard deviation and chemical structure for cellulose diacetate.

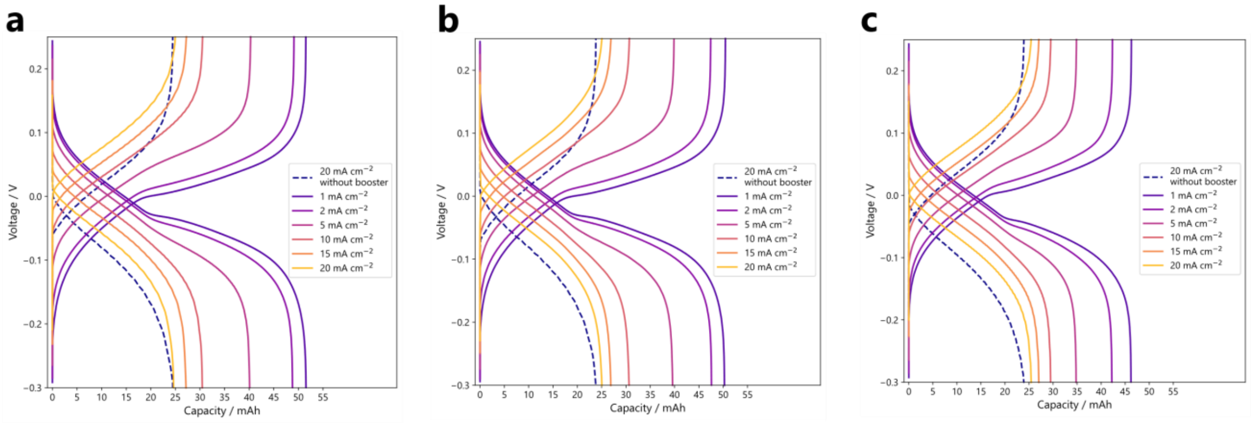

Figure S9. Example of a galvanostatic voltage profile of a symmetric flow battery with 25 mL of 0.1 M $[Fe(CN)_6]^{3-}$ as negolyte and 10 mL of 0.1 M $[Fe(CN)_6]^{4-}$ as posolyte, both in 0.5 M LiCl and 20 vol% DMSO. To establish the baseline capacity of the electrolyte, a galvanostatic cycling step was performed at 20 mA cm^{-2} before addition of **(a)** CA porous, **(b)** PCL porous and **(c)** PVDF porous booster pellets containing one molar equivalent of LFP and cycling at 1 mA cm^{-2}. The 5th cycle of each galvanostatic step is plotted.

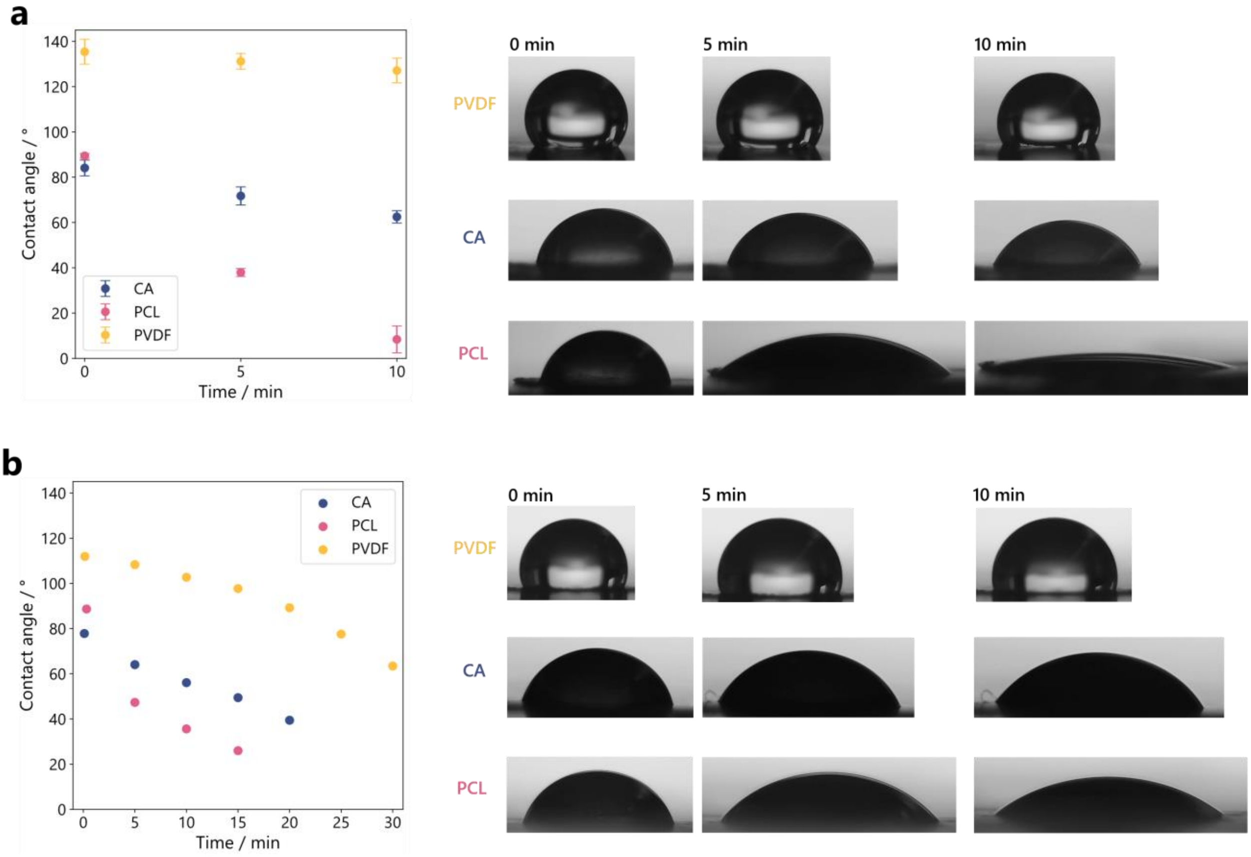

Figure S10. **(a)** Average contact angle values over time for water droplets on LFP-binder composite electrodes (95:5 ratio of LFP:binder). Measurements were performed in triplicate for each electrode composition; standard deviations are shown as error bars. **(b)** Contact angle values over time for droplets of the electrolyte used for the symmetric flow cell tests (0.1 M $[Fe(CN)_6]^{4-}$ in 0.5 M LiCl and 20 vol% DMSO) on LFP-binder composite electrodes (95:5 ratio of LFP:binder).

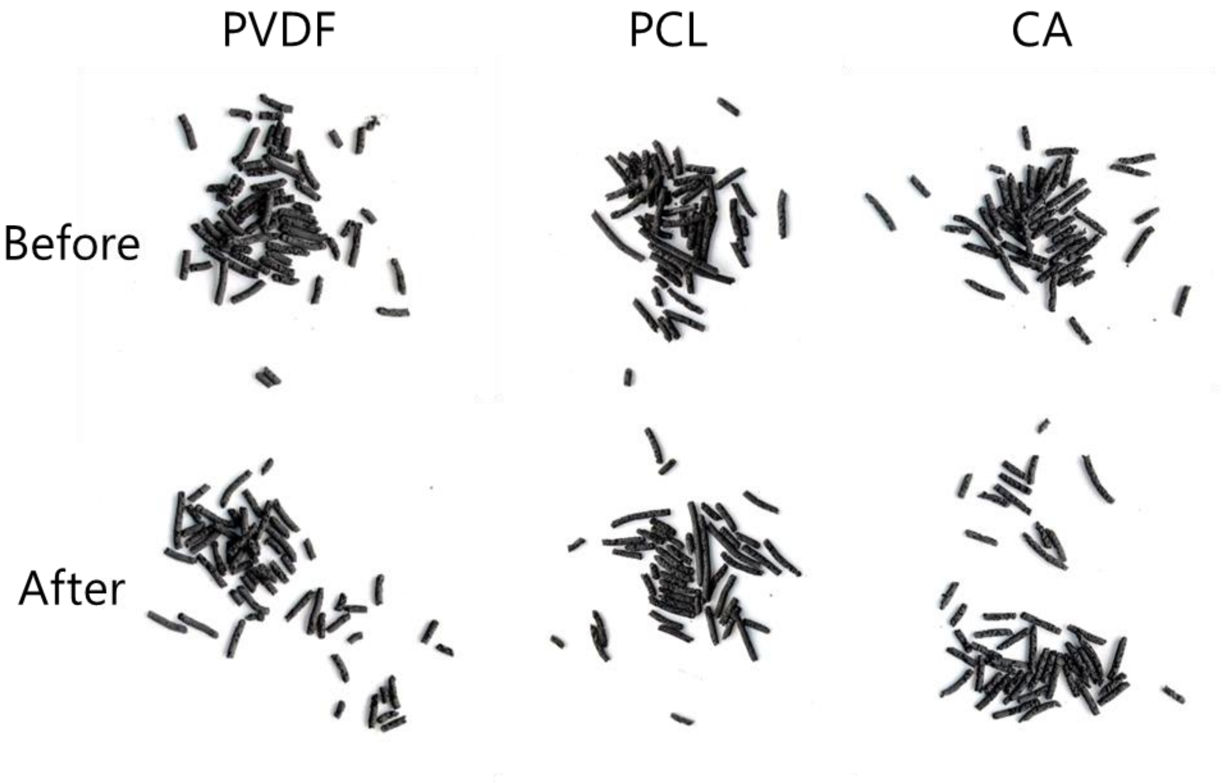

Figure S11. Booster pellets before and after rate tests in symmetric cells.

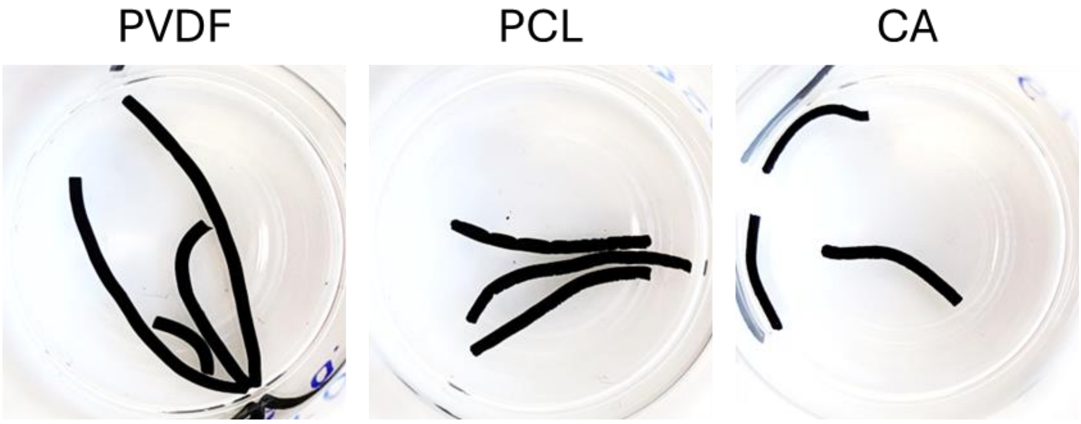

Figure S12. Booster pellets after submersion in ethanol for six months.
